# Regulation of peripheral blood flow in Complex Regional Pain Syndrome: clinical implication for symptomatic relief and pain management

**DOI:** 10.1186/1471-2474-10-116

**Published:** 2009-09-23

**Authors:** George Groeneweg, Frank JPM Huygen, Terence J Coderre, Freek J Zijlstra

**Affiliations:** 1 Department of Anesthesiology, Subdivision Pain Treatment Centre, Erasmus MC, Rotterdam, the Netherlands; 2 Departments of Anesthesia, Neurology, Neurosurgery and Psychology, McGill University, Montreal, Quebec, Canada; 3 Alan Edwards Centre for Research on Pain, McGill University, Montreal, Quebec, Canada; 4 McGill University Health Centre Research Institute, Montreal, Quebec, Canada; 5 Department of Anesthesiology, Subdivision Experimental Anesthesiology, Erasmus MC, Rotterdam, the Netherlands

## Abstract

**Background:**

During the chronic stage of Complex Regional Pain Syndrome (CRPS), impaired microcirculation is related to increased vasoconstriction, tissue hypoxia, and metabolic tissue acidosis in the affected limb. Several mechanisms may be responsible for the ischemia and pain in chronic cold CPRS.

**Discussion:**

The diminished blood flow may be caused by either sympathetic dysfunction, hypersensitivity to circulating catecholamines, or endothelial dysfunction. The pain may be of neuropathic, inflammatory, nociceptive, or functional nature, or of mixed origin.

**Summary:**

The origin of the pain should be the basis of the symptomatic therapy. Since the difference in temperature between both hands fluctuates over time in cold CRPS, when in doubt, the clinician should prioritize the patient's report of a persistent cold extremity over clinical tests that show no difference. Future research should focus on developing easily applied methods for clinical use to differentiate between central and peripheral blood flow regulation disorders in individual patients.

## Background

Complex Regional Pain Syndrome (CRPS) is a painful disorder that usually develops as a disproportionate consequence of trauma. The disorder most commonly occurs in the limbs, and is characterized by spontaneous pain, allodynia and mechanical hyperalgesia, abnormal regulation of blood flow and sweating, oedema of skin and subcutaneous tissues, movement disorders, and trophic changes of skin, organs of the skin, and subcutaneous tissues [1,2].

Growing evidence indicates that CRPS is accompanied by various abnormalities of the microvascular system, including an increase in the number of capillaries [3,4], endothelial swelling, and changes in the vessel luminal wall [5]. These impressive capillary changes range from severely thickened basal membrane with intimal vacuolization, perivascular edema, and debris from pericytes between the basal membrane layers, to necrosis [6,7]. Greatly thickened multi-laminated walls are also observed, which considerably reduce the inner diameter of the vessel [4,8]. Endothelial cells exhibit a shrunken appearance and capillaries with only endothelial cell debris in the lumina have been observed, while other capillaries could be traced by the thickened basal membrane only, lacking the presence of other cellular remnants [7].

In an autopsy study of the affected limbs of two patients, we found an increased number of migrated endothelial cells, as well as an increase of eNOS activity in distal dermis specimens, indicating that endothelial dysfunction may play a role in chronic CRPS [9].

In the CRPS diagnostic criteria [10], a clear distinction is made between two subtypes to reflect the absence or presence of evidence of peripheral nerve injury. However, growing evidence of minor nerve lesions in CRPS [8,11] indicates that this distinction may be artificial.

Although the debate regarding the pathophysiology is still ongoing, the role of excessive regional inflammation, peripheral sensitization of primary somatosensory afferents, and central sensitization of spinal neurons is becoming clear [1,2,12,13]. Recently, evidence was found for the presence of oxidative stress in CRPS patients since they exhibited increases in salivary and/or serum lipid peroxidation products and antioxidants [14,15]. Also recently, Eberle et al. were able to demonstrate differences between warm and cold CRPS including differences in Quantitative Sensory Testing: more prominent sensory loss in cold CRPS and more mechanical hyperalgesia in warm CRPS [16].

The signs and symptoms are related to these mechanisms. Relating the clinical picture to the underlying pathophysiology might help determine the pharmacotherapeutic approach for an individual patient [17].

The clinical picture of CRPS, especially the signs of autonomic dysfunction, and the discovery by Leriche that surgical sympathectomy dramatically improved pain in CRPS supports the important role of the sympathetic nervous system in CPRS etiology [18]. The sympathetic vascular regulatory system in CRPS was extensively examined by Baron et al., who measured differences in blood flow and skin temperature in patients with CRPS after a cold and warm acclimatization period, respectively. The results indicated that differences in skin temperature and blood flow are not static descriptors, but dynamic values mostly dependent on environmental temperature and likely emotional stress [19]. After sympathectomy, in three out of four patients with cold CRPS, the affected limb was considerably warmer and blood flow was considerably higher compared to the healthy side. After a few weeks, however, skin temperature and perfusion slowly diminished, and the affected hand became cold again. Denervation supersensitivity due to complete sympathectomy was thought to be the underlying mechanism of these alterations [19,20]. Wasner et al. measured hand temperature in CRPS patients and healthy control groups while changing whole-body thermal stress using a thermal suit [21]. Whole-body cooling appears to be the most effective way to induce massive tonic activation of cutaneous vasoconstrictor neurons [22]. Three distinct vascular regulation patterns were identified, related to the duration of the disorder. Temperature and blood flow differences between the two sides were dynamic and most prominent at a high to medium level of vasoconstrictor activity [21]. As a result, impairments of thermoregulatory responses should be considered only for diagnostic reasons [23,24].

In the acute phase of CRPS, the affected limb is usually warmer than the contralateral limb due to cutaneous vasodilation, and a functional inhibition of sympathetic vasoconstrictor activity has been shown [21,25]. In this phase, the thermoregulatory blood flow is increased, but the nutritive skin blood flow is unaltered; these differences may be due to differences in regulatory mechanisms. The smooth muscles in the arterioles of the nutritive capillaries are controlled by local factors, whereas the arterioles in the subpapillary plexus are predominantly sympathetically controlled [26,27].

After a so-called intermediate phase in which skin blood flow and temperature differences appear to alternate between warm and cold, a large number of patients show a permanent decrease in blood flow and temperature [28] despite the return of sympathetic vasoconstrictor activity with the duration of the disease [25]. This decrease has been attributed to an increased sensitivity to circulating catecholamines, probably due to upregulation of adrenoceptors following the initial period of reduced sympathetic input [21,26,29-31]. The decrease in blood flow in the intermediate and cold phase occurs in both the thermoregulatory and the nutritive microcirculation [26].

As demonstrated by simultaneous testing of skin blood flow and sweat in one CRPS patient, vascular abnormalities may not solely be the result of a disturbance of the autonomic nervous system. Although sudomotor activity was greater on the affected side, implying higher sympathetic nerve activity, basal blood flow was also greater on the same side. These results suggest that one or more factors increase basal blood flow despite high sympathetic nerve tone on the affected side [32]. Other mechanisms may also be involved in the pathophysiology of the vascular abnormalities. The demonstrated increase in vasoconstriction, tissue hypoxia, metabolic acidosis [33,34] and vascular permeability for macromolecules [35] may also indicate endothelial dysfunction.

Several recent studies [36-40] have demonstrated alterations in the central sensorimotor processing in CRPS patients. Changes in the central nervous system [41] play a very important role in the current conception of CRPS pathophysiology as well as its treatment. This paper, however, concentrates on the central and peripheral mechanisms responsible for vascular abnormalities and pain in CRPS; furthermore, we suggest potential treatment approaches for CPRS.

## Discussion

### Mediators of changes in blood flow

The endothelium, the largest organ of the body, releases agents that regulate vasomotor function, trigger inflammatory processes and affect hemostasis in response to shear stress and hormonal stimuli, such as vasoactive substances [42]. Blood pressure and blood flow is regulated by the release of the vasodilators nitric oxide (NO) and prostacyclin (PGI_2_), and the vasoconstrictor endothelin (ET) [43].

NO is generated from the amino acid L-arginine by three major isoforms of NO synthase, namely neuronal (nNOS), inducible (iNOS), and endothelial (eNOS) enzymes [44]. nNOS and eNOS are constitutively expressed and activated by calcium entry into the cells; iNOS is calcium independent, and its synthesis is induced in inflammatory and other cell types by stimuli such as endotoxin and proinflammatory cytokines [44]. NO diffuses through the artery wall to the vascular smooth muscle cells in the media, where it increases the activity of guanylate cyclase and the concentration of cyclic guanosine monophosphate (cGMP), thus relaxing the vascular smooth muscle and leading to vasodilation [45]. PGI_2_, which only plays a limited role as a vasodilator in most vascular beds, is produced from arachidonic acid by cyclooxygenase in response to shear stress and a number of factors that also increase NO production. PGI_2 _activates adenylate cyclase to increase cyclic adenosine monophosphate (cAMP), also leading to vasodilation [46,47].

The peptide ET, one of the most potent known vasoconstrictors, is generated by cleavage of a large polypeptide within the endothelium. Of the three types of ET, ET-1 is the most important in vascular tissue, and it acts on endothelin-A receptors on the vascular smooth muscle [48]. Evidence suggests a feedback mechanism between ET and NO, as ET inhibits the production of NO, while NO inhibits ET production [44,47,49,50]. The major biological effects of these vasoactive substances depend on their rapid synthesis. The maximal NO stimulation can be reached within seconds, but the maximal vasoconstrictive response of endothelium *in vivo *takes up to one hour [51]. Vasomotor tone is regulated by ET-1 and NO, depending on the health of the endothelium [51,52].

### Endothelial dysfunction

Endothelial dysfunction (EtD) was first described by Panza as an impairment of the regulation of endothelium on blood vessels [53]. As a proinflammatory and prothrombic state, EtD has been described in the pathophysiology of different forms of cardiovascular disease, including hypertension, coronary artery disease, chronic heart failure, peripheral artery disease, diabetes, and chronic renal failure [42]. Under these conditions, the levels of pro-oxidant reactive oxygen species in the vessel wall are elevated. NO is rapidly degraded by oxidant stress and the vasoinactive and toxic peroxynitrite is produced. In both animal and human studies, this process could be prevented by administration of high concentrations of the antioxidant vitamin C [51,54,55].

Flow mediated dilation (FMD) is an important tool for assessment of endothelial function both in the upper [56] and the lower [57] limb. *In vivo*, endothelial function may be assessed invasively on resistance arteries by measuring blood flow using strain-gauge plethysmography by studying the effects of acetylcholine or metacholine administration through an intra-arterial catheter [58]. Acetylcholine has a direct vasoconstrictive effect on vascular smooth muscles. However, NO from activated healthy endothelium overwhelms the direct effect of acetylcholine, which results in vasodilation. This vasodilation is blocked by inhibitors of NO synthesis, such as monomethylarginine. In endothelial dysfunction, less NO is generated, which may ultimately lead to a vasoconstrictive response to acetylcholine [51,59,60]. Shear stress, which stimulates the endothelium to release NO, may be used to non-invasively induce reactive hyperaemia, and the subsequent changes in blood flow can be measured by ultrasound. This has been shown in the brachial artery using FMD [61]. Other non-invasive *in vivo *techniques include fingertip pulse wave amplitude with peripheral arterial tonometry [62], measurement of intima-media thickness [63], and flow waveform patterns [64]. *In vitro*, the endothelial dysfunction of isolated resistance arteries dissected from biopsies of gluteal subcutaneous tissue has been studied on a wire or pressurized myograph. A good correlation was found between the endothelial function of small arteries *in vitro *and FMD of the brachial artery *in vivo *[42,65].

Several studies have assessed the microvascular endothelial function in CRPS patients with controversial results. Gorodkin et al. used iontophoresis to test endothelial dependent vasodilation with acetylcholine and endothelial independent vasodilation with sodium nitroprusside in 17 CRPS patients and 16 healthy controls. Among the CRPS patients (IASP criteria) 4 were qualified as warm and 13 as cold; the median duration of the symptoms was 5 years (range 3 months to 20 years). No significant differences were observed between both groups or between affected and unaffected limbs [66].

However, these results could not be reproduced by Schattschneider et al., who compared 14 patients with cold CRPS (disease duration 17.6 ± 2.1 months) with 10 healthy controls. In this study, acetylcholine induced vasodilation was significantly reduced on the affected side compared to the contralateral extremity and controls, whereas no differences were observed after application of sodium nitroprusside [67].

Similar results were found in a study by Duman et al., who used the FMD technique to examine 21 patients in a more acute stage of CRPS (IASP criteria, disease duration 5.9 ± 2.5 months), and compared the results with 15 healthy controls. Upon evaluation of the brachial artery with Doppler ultrasound, significant differences were observed in the waveforms obtained in the affected compared to contralateral limbs; although not significant, there was a trend of larger dilating responses in the affected limbs [64].

In a small study with 9 patients with CRPS in one upper limb and 9 patients with CRPS in a lower limb, Dayan et al. examined FMD and local vascular reflexes [68]. To measure the venoarteriolar reflex (VAR), a cuff is inflated to a steady pressure of 40 mm Hg for 4 minutes to produce venous congestion, which causes a reflexive regional arteriolar vasoconstriction. To measure the microvascular myogenic reflex (VMR), the subject is placed in supine position to avoid systemic baroreflex changes and the leg or forearm is measured during 40 cm dependency of the leg or forearm below cardiac level for 4 minutes. This activates the myogenic response by increasing the arterial wall pressure, and the venoarteriolar response by gravitationally increasing the venous pressure. The result is local vasoconstriction and, consequently, a reduced blood flow. The duration of the disease was 40 and 46 months for upper and lower limb CRPS, respectively, which was diagnosed using the IASP criteria. In comparing the affected limb to the contralateral side, the resistance artery FMD was impaired on the CRPS side along with exaggerated arteriolar vasoconstriction following activation of the VAR, while the VMR remained unchanged. These changes in vascular reflexes were only significant in the lower limbs. The VAR depends mostly upon intact local autonomic nervous functions, but the VMR is an inherent arteriolar muscle constriction reflex in response to dilation, which appears to be independent of neural transmission. Based on these observations, the authors concluded that the impaired VAR and intact VMR might reflect the adrenergic hypersensitivity in the lower limbs in patients with CRPS [68].

The results from these four studies suggest that changes in vascular response are found locally only at the side affected by CRPS, while there is no difference between the contralateral side and healthy controls. Whether there is a relation between stage of the disease (warm, intermediate or cold) or the duration of the disease, and endothelial dysfunction is not yet clear. In discussing the first three trials, Duman [64] suggested that symptoms of vascular changes, such as hyperemia and edema, may lose their prominence in chronic CRPS, whereas they may be more evident in earlier stages. On the other hand, the study by Dayan in a group of patients with longer disease duration showed clear differences. No significant differences in blood flow were found between CRPS side, unaffected side or healthy controls at baseline. This is another indication that the regulatory mechanisms in particular are affected in vascular alterations in CRPS. The same conclusion was previously made by Wasner [21], who studied the sympathetic regulation mechanism in the hands of CRPS patients by inducing temperature changes to the entire body with a thermal suit; they concluded that the differences in blood flow and temperature were not static. No significant differences were detectable during low or absent sympathetic vasoconstrictor activity, and these differences were most pronounced during periods of intermediate to high sympathetic activity [21].

In addition to changes in the somatosensory systems, which process noxious, tactile and thermal information, and changes to the sympathetic systems, which innervate skin and somatomotor systems, peripheral changes are detected in CRPS that cannot be explained by the central changes [1]. These peripheral changes (sympathetic afferent coupling, vascular changes, inflammatory changes, edema, and trophic changes) cannot be seen independent of the central changes [1,69]. Furthermore, each symptom can also be generated by more than one mechanism, depending on the patient. Therefore, caution must be taken when grouping patients based on symptoms and administering drugs irrespective of their underlying disease [1]. A cold extremity in chronic CRPS could be caused by several factors, such as increased tone of the sympathetic nervous system, pathologic alterations of the vascular wall, changes in small nerve fibers innervating the blood vessels, or endothelial dysfunction. It is also possible that as symptoms of endothelial injury progress, there may be a shift from a predominant oedema associated with plasma extravasation from damaged post-capillary venules (an early consequence of endothelial injury) to chronic ischemia with the development of arterial vasospasms and capillary no-reflow (later consequences of endothelial injury). Furthermore, sympathetic blocks or the use of vasodilatory agents may overcome ischemia that is dependent on arterial vasospasms, which represents a functional reduction in blood flow; this, however, would not be able to overcome ischemia associated with capillary no-reflow, which represents a physical reduction in blood flow. These observations may explain why the results of trials studying medication to improve blood flow in patients with cold chronic CRPS do not show more convincing results. It may also explain, why an intervention to improve blood flow does not improve pain in all patients alike.

### Pain

Pain is an important symptom in CRPS. Most patients experience an intense, spontaneous, burning pain in the distal part of the affected limb, which is characteristically disproportionate in intensity to the initial event, and increases in a dependent position [1]. Several types of pain can be distinguished: neuropathic, inflammatory, nociceptive and functional [70]. Evidence supports a neuropathic origin of the pain in CRPS [71]. The spatial distribution of pain and sensory abnormalities such as allodynia to mechanical, cold and heat stimuli, as well as hyperalgesia, indicate that the pathophysiological mechanisms in CRPS involve both the peripheral and central nervous system [1,72,73], but the interaction between the peripheral and central changes is only partially understood [71].

The various mechanisms by which pain is generated in CRPS are outlined below. The section on ischemic pain is of particular relevance to this discussion, as it closely relates to changes in peripheral blood flow.

### Neuropathic pain

The neuropathic pain in peripheral tissues may be generated and maintained by peripheral sensory nerve fibers. Quantitative sensory testing studies strongly suggests a role of peripheral inflammation in acute CRPS, and a loss of small fibres in acute as well as chronic CRPS. Acute CRPS patients demonstrated heat pain hyperalgesia, while chronic CRPS (>12 months) showed warm as a well as cold hypoesthesia [40]. Oaklander et al. analyzed the innervation density of the epidermis, and their results suggest that CRPS is especially associated with persistent minimal distal nerve injury affecting nociceptive small fibers, a type of nerve injury that will remain undetected in most clinical settings [11]. Albrecht studied the innervation of CRPS affected skin tissue, and found impressive changes in innervation of different target tissues as well as changes of the target tissue itself (e.g. blood vessels) [8]. Although the results of these two studies must be interpreted with care, as these observations may be the result of secondary tissue changes that may occur in the course of the disease, both studies indicate that CRPS can be associated with peripheral pathological changes of innervation of the skin, thus CRPS may indeed be a neuropathic pain syndrome [8,11,71]. Neuropathic pain may also be caused by aberrant activity of the sympathetic nervous system (SNS), in which case the pain is referred to as sympathetically maintained pain (SMP) [72,74]. In patients with SMP, sympathetic blockade relieves spontaneous pain and mechanical hyperalgesia, but these symptoms may reappear following intracutaneous application of noradrenaline [19,75,76] or by stimulation of the SNS by cooling of the body or forehead, or a startle stimulus [77,78]. SMP is not typically observed in CRPS; only 50% of CRPS patients experience SMP [79-81], and a number of neuropathic pain syndromes might also benefit from sympathetic blocks [30,72]. It should be noted, that the Cochrane analysis states that 'no conclusion concerning the effectiveness of sympathetic blocks could be drawn' [82].

### Inflammatory Pain

Sudeck was the first to describe the classic signs and symptoms of inflammation, including rubor, calor, dolor, tumor and function laesa, in acute CRPS [83,84]. Kozin [85] described inflammatory changes in 2 patients with early CRPS, and Oyen [35] found increased vascular permeability for macromolecules, which was thought to be due to the inflammatory response, caused by free oxygen radicals. Increased systemic calcitonin gene-related peptide (CGRP) serum concentrations were found by Birklein [86], suggesting neurogenic inflammation as the pathophysiologic mechanism. In this study, however, pain and hyperalgesia were observed in chronic stages in particular, independent of the increased neuropeptide concentration.

Our research group compared levels of proinflammatory cytokines tumor necrosis factor alpha (TNF-α) and interleukin 6 (IL-6) in plasma and fluids of artificially induced blisters on the CRPS side and contralateral side. There was no indication for inflammation in plasma [12], which was confirmed by van de Beek [87]. However, Huygen et al. demonstrated that TNF-α and IL-6 levels were increased in blister fluids in patients with acute CRPS [12]. This shift in the pro-inflammatory cytokine profile in acute CRPS patients was also found by Uceyler [2].

In three studies of patients with an intermediate disease duration of 2.5, 2.8 and 3.5 years, the levels of these cytokines were still increased [88-90]. Remarkably, a large proportion of these patients no longer displayed any of the above-mentioned signs and symptoms of inflammation. A study of chronic CRPS patients with a disease duration of 6 years showed no differences in TNF-α and IL-6, although some patients still showed signs of inflammation [91]. No relationship between pro-inflammatory cytokines and disease characteristics, such as pain, changes in temperature, volume, mobility and disease duration, was observed in either study [88,91].

### Ischemic pain

In rats, during the reperfusion which follows prolonged extremity ischemia, the synthesis of free radicals and pro-inflammatory cytokines leads to inflammatory responses and vasculature injury in the ischemic tissue [92]. This ischemia-reperfusion causes damage to the endothelial cells, resulting in swelling and protrusion of cells in the capillary lumen with impeded passage of red blood cells as consequence, the so-called slow-flow/no-reflow phenomenon. Coderre also showed that ischemia-reperfusion of the rat hind paw induced long-term mechanical and cold hypersensitivity, which was effectively reduced by free radical scavengers [93] as well as classical analgesics, although to a lesser extent [94]. These symptoms are comparable to those described in humans with CRPS. Recently, Coderre and colleagues also showed that mechanical allodynia induced by hind paw ischemia-reperfusion injury is accompanied by increased hind paw muscle malondialdehyde (a product of free radical-induced lipid peroxidation), pro-inflammatory cytokines (IL-1β, IL-6 and TNFα), nuclear factor κB and lactate; furthermore, mechanical allodynia is reduced by inhibitors of these mediators or an antagonist at acid sensing ion channels (ASICs) [95]. The authors also demonstrated that mechanical allodynia following ischemia-reperfusion injury parallels the development of arterial vasospasms, endothelial cell thickening and capillary slow-flow/no-reflow in hind paw muscle, and is directly correlated with muscle lactate but not with the demonstrated reduction in intraepidermal nerve fibers [95,96]. Furthermore, similar to CRPS patients, rats with hind paw ischemia-reperfusion injuries exhibit enhanced pain and allodynia following exercise, symptoms that depend on increases in muscle lactate [95].

In human patients treatment with free radical scavengers can both reduce the risk of developing CRPS [97-100] and improve the clinical picture [66,101-103]. This observation was supported by recent evidence of increased levels of saliva and serum antioxidants and serum malondialdehyde in CRPS patients [14], which is almost definitive proof for the involvement of free radicals in the pathophysiology.

Regardless of the initial pathogenesis, one hypothesis has proposed a vicious circle of altered blood flow that leads to hypoxia, production of free radicals, endothelial damage and further reductions in blood flow [66]. The slow-flow/no-reflow injury may even affect the microvasculature of peripheral nerves, thus presenting the cause of peripheral neuroinflammation in CRPS [15].

### Nociceptive pain

One of the characteristics of CRPS is the often observed disproportionate severity of the symptoms with the severity of the trauma, along with a tendency to generalize in the affected distal limb but not confined to the innervation zone of an individual nerve [30]. Nociceptive pain may be caused by tissue damage as a result of the initiating trauma or by secondary tissue changes that occur in the course of the disease, i.e. oedema, changes in the nutritive blood flow, hypoxia, lactate increase and acidosis [33,34,71,104-106].

### Functional pain

Functional pain is defined as hypersensitivity to pain resulting from abnormal central processing of normal input [70]. CRPS patients are known to protect their involved limb to minimize pain associated with movement and touching; this protection has been described as a voluntary action, but lately neglect-like behavior has been proposed to play an important role. Two terms have been introduced in describing this behavior: 'cognitive neglect', which suggests that patients perceive their involved limb as feeling foreign to them, and 'motor neglect', which describes situations in which the patients need to focus mental and visual attention to move their limb [107]. In a survey of 224 CRPS patients, 84% of the respondents confirmed the presence of at least one of these neglect symptoms, and 47% indicated they experienced both [107]. Other studies also found a large proportion of CRPS patients with disturbances of self-perception of hand or foot, indicating an alteration in the processing of the higher central nervous system [108-110].

Disuse has often been mentioned in CRPS. In animal studies of immobilization, an increased sensitivity to sensory stimuli, as well as changes at the spinal level that could account for this increase, have been found [111-113]. In humans, casting produced increased cerebral blood flow in areas associated with sensory processing, motor function, and emotions [112,114]. These studies indicate that immobility alone may produce many signs and symptoms also found in patients with CRPS [112]. Fear of injury has also been suggested as a potential predictor of disability in CRPS [115], and combined with increased sensitivity for pain, fear of injury may lead to excessive guarding and over-protective behaviors [116]. Rommel et al. found that depressive syndromes frequently develop with chronic CRPS, and psychological treatment can be recommended [117]. Nevertheless, despite the often suggested relationship between CRPS and a psychological predisposition, Van de Laan et al. used the SCL 90 and did not find any specific psychological profiles in CRPS-dystonia [118]. In a large population-based study, De Mos et al. also did not find any relationship between pre-existing psychiatric disorders and CRPS (odds ratio of 1.17) [119].

### Pharmalogical interventions

We investigated the involvement of the vasoactive substances ET and NO during early chronic CRPS. Measurement of NO and ET levels in artificial suction blisters in 29 patients showed a significant increase in ET and a decrease of NO on the affected side compared to the unaffected side, indicating an aberrant NO/ET ratio in the intermediate state of CRPS. This altered ratio results in vasoconstriction and consequently in diminished tissue blood distribution [89]. The ratio could be restored by the substitution of NO, substitution of asymmetric dimethylarginine (ADMA) [120], or the blocking of ET_A _receptors [49]. As several recent publications indicate that NO substitution might be valuable in treating diabetic neuropathy [121], anal fissures [122,123], and epicondylitis [124], we propose that the effective treatment of CRPS by NO substitution, which was shown previously [125], occurs by increasing blood flow. In a pilot study, five female patients with cold type CRPS in one hand were treated with NO donor isosorbide dinitrate (ISDN) ointment 4 times daily for 10 weeks; videothermography was used to monitor changes in blood distribution in both the involved and contralateral extremities. The patients treated with ISDN showed a 4-6°C increase in mean skin temperature of the cold CRPS hands, reaching temperatures similar to that of contralateral extremities within 2-4 weeks, suggesting normalization of blood distribution; normalization was confirmed by an improvement in skin color. In three patients, the VAS pain declined, whereas in the other two patients, the VAS pain remained unchanged. Thus, the topical application of ISDN appeared to be beneficial in improving symptoms for patients with cold type CRPS [126].

**Figure 1:**
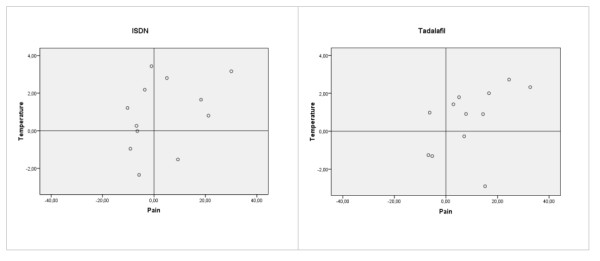
**Relationships between the improvements in temperature and pain in the ISDN and Tadalafil studies**. The improvement in pain is shown on the horizontal axis. The Visual Analogue Scale (VAS, score 0-100) is the average of actual pain scores that were recorded three times each day (0800, 1200 and 2000) during one week before the first and the last hospital visits. The improvement in pain is calculated by subtracting the VAS score at the end of the study from the VAS score at the start of the study; thus, a positive value indicates less pain. The vertical axis shows the changes in temperature calculated by subtracting the temperature difference (CRPS side minus contralateral side) between the dorsal side of both hands at the end of the study from the difference measured at the start of the study. A positive value indicates that the difference in temperature has diminished.

Based on these preliminary results, we decided to test the effect of ISDN in a double blind randomized controlled trial. Twenty-four patients with chronic CRPS in one upper extremity received 1% ISDN in Vaseline or a placebo ointment applied to the dorsum of the affected hand 4 times daily for 10 weeks. The patients participated in a physical therapy program to improve activity. The primary outcome measure was blood distribution in the affected extremity, which was determined by measuring the skin temperature using videothermography. We also measured NO and ET-1 concentrations in blister fluid, assessed pain using the VAS, and determined activity limitations using an Upper Limb Activity Monitor (ULAM) and the Disabilities of Arm Shoulder and Hand Questionnaire (DASH). ISDN failed to produce a significant improvement in temperature asymmetry in chronic cold CRPS patients, and also did not generate the predicted reduction in pain and increase in activity compared to placebo. The results of the active treatment group are shown in Figure 1. Together this suggests that other central or peripheral factors may contribute to the disturbed vasodynamics in cold chronic CRPS that are not influenced by NO substitution [127].

To further evaluate the influence of endothelial factors, patients with chronic cold CRPS in one lower extremity were included in a double blind, randomized, controlled trial that investigated the effect of tadalafil on the microcirculation. Tadalafil, a PDE-5-inhibitor known as an effective treatment for erectile dysfunction [128], functions within the vascular smooth muscle cell to inhibit the hydrolyzation of cyclic guanosine monophosphate (cGMP) to GMP. Through the phosphorylation of specific proteins and ion channels, treatment with tadalafil results in the opening of potassium channels and hyperpolarization of the muscle cell membrane, sequestration of intracellular calcium by the endoplasmic reticulum, and block of calcium influx by the inhibition of calcium channels. The consequence is a drop in cytosolic calcium concentrations and relaxation of the smooth muscle that causes vasodilation [128]. In this trial, twenty-four patients received 20 mg tadalafil or placebo daily for 12 weeks, and participated in a physical therapy program. The primary outcome measure was temperature difference between the CRPS and the contralateral sides, as determined by measuring the skin temperature with videothermography. Secondary outcomes were pain measured on a VAS, muscle force measured with a MicroFet 2 dynamometer, and level of activity measured with an Activity Monitor (AM) and walking tests. At the end of the study period, the temperature asymmetry was not significantly reduced in the tadalafil group compared with the placebo group, but there was a significant and clinically relevant reduction of pain in the tadalafil group. Muscle force improved in both treatment groups, and the AM revealed small, non-significant improvements in time spent standing and walking, as well as the number of short walking periods. The results of the active treatment group are also shown in Figure 1. Thus, tadalafil may be a promising new treatment for patients with chronic cold CRPS. The use of tadalafil and the role of endothelial dysfunction in CRPS warrants further investigation [129].

In both studies, the results of treatment with study medication was compared to placebo, and, as shown in Figure 1, there were clear responders and non-responders among the patients that received the active medication in both studies.

The results of the static videothermographic measurements should be interpreted with care, due to the dynamic nature of side temperature differences in CRPS [19,21,130,131]. Long-term skin temperature measurements may prove to be a more reliable instrument for determining temperature changes in CRPS [132]. Furthermore, care should be taken when using the clinically unaffected side as a control for studies on thermoregulatory skin blood flow in the CRPS side. One possibility is that the thermoregulatory skin blood flow in both extremities may have resulted from a spinal reflex mechanism initiated by (post-) traumatic excitation of a peripheral nerve on the clinically affected side [133]. Given the pathology of muscle tissue (including lipofuscin deposits, atrophic fibers, and severely thickened capillary basal membranes) observed in the amputated limbs of CRSP patients as described by van der Laan et al. [7], as well as the role of muscle pathology (increased lipid peroxidation products, pro-inflammatory cytokines and lactate) in mechanical allodynia of rats with ischemia-reperfusion injury, alterations in deep tissue blood flow, as well as skin blood flow, may be important in CRPS. Also, since capillary hemoglobin oxygenation (HbO_2_) is lowered and skin lactate is increased in CRPS limbs, alterations in nutritive as well as thermoregulatory blood flow may also be important in CRPS.

Recent evidence suggests that both NO and ET also play a role in nociception exhibited in animals with ischemia-reperfusion injuries. The mechanical allodynia that is observed in rats with a hind paw ischemia-reperfusion injury is relieved by systemic administration of the NO donor 3-morpholinylsydnoneimine chloride (SIN-1) [96]. Furthermore, intradermal injections of either norepinephrine or the endothelial NO synthase inhibitor N5-(1-Iminoethyl)-L-ornithine dihydrochloride (L-NIO) (both of which should reduce blood flow) induce sustained nociceptive behaviours in rats with hind paw ischemia-reperfusion injuries; the nociceptive behaviours induced by NE are reduced by local or systemic administration of the NO donors sodium nitroprusside and SIN-1, respectively [96]. This observation raises the possibility that sympathetically maintained pain may depend more on NE-induced vasoconstriction than on sympathetic-afferent coupling. This conclusion is supported by the finding that sustained nociceptive behaviours are also induced in rats with hind paw ischemia reperfusion injuries after intradermal injection of the non-adrenergic vasoconstrictor vasopressin [96]. Coderre and colleagues recently demonstrated that mice with ischemia-reperfusion injury of the hind paw exhibit sustained nociceptive behaviors following intradermal injection of ET-1 or ET-2. These nociceptive behaviours correlated with increased ET-A receptor expression in hind paw muscle, and were reduced by co-administration of an ET-A antagonist (T. Coderre, personal communication).

Treatment strategies for endothelial dysfunction in CRPS patients include the substitution of nitric oxide with ISDN, inhibition of PDE5, substitution of ADMA, or blockage of ET_A _receptors. Of these strategies, only ISDN and PDE5 inhibition have been tested in randomized placebo-controlled trials with CRPS patients. Because these trials included patients with cold chronic CRPS and did not differentiate between central and peripheral dysfunction, the results may not be conclusive for the treatment of endothelial dysfunction.

Calcium antagonists are used in the treatment of hypertension and angina pectoris, as they bind to the L-type calcium channel in the smooth muscles of the vascular wall, thus reducing the influx of extracellular calcium and resulting in vasodilation. Nifedepine has been examined in two descriptive studies with CRPS patients, and this calcium antagonist was most effective in acute CRPS [134,135]. In two studies performed by our research group, only a few patients used the calcium antagonist verapamil and none used nifedepine. In addition to peripheral vasodilation, verapamil also causes a reduction of the heart rate and atrioventricular conduction. Since nifedepine is a more potent peripheral vasodilator with only minimal cardiac side-effects [136], one of the long-acting dihydropyridines such as nifedipine might prove advantageous in cold chronic CRPS [137]. More research on this subject is warranted.

An interesting development occurred in the tadalafil study, when one patient who had not responded to the active tadalafil treatment with an increase of temperature and showed persistent high pain scores was treated with a lumbal sympathectomy. The leg on the CRPS side became warmer, and the temperature increased until it was more than 1°C warmer than the contralateral side. This improvement in temperature (difference) still persists. We consider this an indication that in cold chronic CRPS, both endothelial dysfunction and sympathetic dysfunction may be responsible for ischemia in the cold extremity. Unfortunately, in this patient, the severe pain was unaffected by the sympathectomy, indicating that in CRPS, pain and ischemia may be related in some cases, but an increase in blood flow will not result in less pain in other cases.

This observation confirms the result of trials performed by Baron et al. in analyzing skin blood flow demonstrating that sympathetic vasoconstrictor reflexes and pain after surgical sympathectomy show no clear relationship of vascular changes and the success of sympathectomy regarding pain relief [19]. Thus, apart from nociceptive and neuropathic pain, pain in chronic cold CRPS may also be due to ischemic pain caused by endothelial dysfunction [67,73,93], sympathetic hyperactivity or increased sensibility to circulating catecholamines [20], or sympathetically maintained pain [77].

In a study with intermediate CRPS patients (disease duration 2.8 ± 1.4 years), we found a significant increase in IL-6, TNF-α and ET-1 levels in blister fluid in the CRPS extremity versus the contralateral extremity [89]. ET-1 concentrations in the cold chronic patients in the ISDN study were lower than those in our previous study, but still higher than levels previously reported by others [127,138,139]. Apparently, some of these chronic cold patients still had active inflammatory components, which may explain the case of one of the outpatients who was treated with a PDE5-inhibitor for a very cold painful foot in chronic CRPS. In a few days, the affected foot displayed full-blown warm CRPS. The classical signs of inflammation (rubor, calor, dolor, tumor and functio laesa) depend highly on unimpaired circulation. Similar to the implications of the ET-1 measurements of the ISDN study, this case suggests that there may be patients with chronic cold CRPS with active inflammation who do not show symptoms of inflammation because of impaired vasodilation. Indeed, it has been shown that plasma extravasation does not occur in the later stages of ischemia-reperfusion injury after the development of no-reflow. Thus, oedema only occurs with leakage of plasma from the post-capillary venules of vessels that are adequately perfused [140]. This may account for persistent pain and other therapy-resistant symptoms in some patients.

Current research in the field suggests several possible mechanisms responsible for ischemia and pain in chronic cold CRPS. Because of the fluctuation of temperature difference between both hands, even a patient who reports an extremely cold extremity may present no difference in temperature in the clinic or even a warmer CRPS side. In this case, a static video thermographic recording may not show any difference compared to a manual test. Under circumstances of doubt, the clinician should let the patient's report of a persistent cold extremity prevail over clinical tests that show no difference.

Several tests have been described to separately investigate the sympathetic and the endothelial function. Until now, these tests have only been used for research purposes. Promising results were found using provocative manoeuvres, like the Valsalva manoeuvre, and the cold pressor test [141], or inspiratory gasp and contralateral cooling [19,142,143].

Future research should focus on methods to differentiate between sympathetic and endothelial dysfunction for clinical use. Laser Doppler Flow, video thermographic recordings and ultrasound could be used to measure the results of autonomic provocation tests, and flow mediated dilation, in combination with long-term temperature measurements. Investigators should not only focus on blood flow in the skin but also in deep tissues; more information is needed regarding the separate contributions of thermoregulatory and nutritive blood flow.

To differentiate between sympathetically maintained pain (SMP) and sympathetically independent pain (SIP), a sympathetic block has been advised [76], usually indicating an invasive chemical or a surgical block [144-147]. Bolel et al., however, described a non-invasive stellate ganglion blockade using diadynamic current [148] that could provide a simple and easy to perform method to differentiate between SMP and SIP.

## Summary

As a result of an impressive amount of research over the last few years, our understanding of CRPS has improved considerably. Although some studies have suggested that CRPS is primarily a disease of the central nervous system, alterations have also been found in the peripheral small nerve fibers innervating skin, blood vessels and sweat glands, as well as aberrations in the vascular wall, muscle fibers and other deep somatic tissues, likely as a result of damage by the initial inflammation. Although this article focused on disturbed peripheral blood flow in patients with cold, chronic CRPS, treatment goals are not necessarily only local. In this respect, we agree with Harden, who proposes that the treatment of CRPS should be a systematic and coordinated interdisciplinary approach with a primary goal of functional restoration [69].

## Competing interests

The authors declare that they have no competing interests.

## Authors' contributions

All authors contributed equally to this article. All authors read and approved the final manuscript.

## Pre-publication history

The pre-publication history for this paper can be accessed here:



## References

[B1] Janig W, Baron R (2003). Complex regional pain syndrome: mystery explained?. Lancet Neurol.

[B2] Uceyler N, Eberle T, Rolke R, Birklein F, Sommer C (2007). Differential expression patterns of cytokines in complex regional pain syndrome. Pain.

[B3] Kozin F, McCarty DJ, Sims J, Genant H (1976). The reflex sympathetic dystrophy syndrome. I. Clinical and histologic studies: evidence for bilaterality, response to corticosteroids and articular involvement. The American journal of medicine.

[B4] Basle MF, Rebel A, Renier JC (1983). Bone tissue in reflex sympathetic dystrophy syndrome--Sudeck's atrophy: structural and ultrastructural studies. Metabolic bone disease & related research.

[B5] Lagier R (1983). Partial algodystrophy of the knee. An anatomico-radiological study of one case. J Rheumatol.

[B6] Kirsch K (1978). [The Sudeck-Leriche syndrome as a disturbance in distant regions of the body, clinical picture, and histology (author's transl)] Das Sudeck-Syndrom als Fernstorung. (Klinik und Histologie). Z Orthop Ihre Grenzgeb.

[B7] Laan L van der, ter Laak HJ, Gabreels-Festen A, Gabreels F, Goris RJ (1998). Complex regional pain syndrome type I (RSD): pathology of skeletal muscle and peripheral nerve. Neurology.

[B8] Albrecht PJ, Hines S, Eisenberg E, Pud D, Finlay DR, Connolly MK, Pare M, Davar G, Rice FL (2006). Pathologic alterations of cutaneous innervation and vasculature in affected limbs from patients with complex regional pain syndrome. Pain.

[B9] Groeneweg JG, Heijmans Antonissen CH, Huygen FJ, Zijlstra FJ (2008). Expression of endothelial nitric oxide synthase and endothelin-1 in skin tissue from amputated limbs of patients with complex regional pain syndrome. Mediators Inflamm.

[B10] Harden RN, Bruehl S, Stanton-Hicks M, Wilson PR (2007). Proposed new diagnostic criteria for complex regional pain syndrome. Pain medicine (Malden, Mass).

[B11] Oaklander AL, Rissmiller JG, Gelman LB, Zheng L, Chang Y, Gott R (2006). Evidence of focal small-fiber axonal degeneration in complex regional pain syndrome-I (reflex sympathetic dystrophy). Pain.

[B12] Huygen FJ, De Bruijn AG, De Bruin MT, Groeneweg JG, Klein J, Zijlstra FJ (2002). Evidence for local inflammation in complex regional pain syndrome type 1. Mediators Inflamm.

[B13] Birklein F (2005). Complex regional pain syndrome. J Neurol.

[B14] Eisenberg E, Shtahl S, Geller R, Reznick AZ, Sharf O, Ravbinovich M, Erenreich A, Nagler RM (2008). Serum and salivary oxidative analysis in Complex Regional Pain Syndrome. Pain.

[B15] Coderre TJ, Bennett GJ (2008). Objectifying CRPS-I. Pain.

[B16] Eberle T, Doganci B, Kramer HH, Geber C, Fechir M, Magerl W, Birklein F (2009). Warm and cold complex regional pain syndromes: differences beyond skin temperature?. Neurology.

[B17] Okudan B, Celik C (2006). Determination of inflammation of reflex sympathetic dystrophy at early stages with Tc-99m HIG scintigraphy: preliminary results. Rheumatology international.

[B18] Leriche R (1916). De la causalgie envisagée comme une névrite du sympathique et de son traitement par la dénudation et l'excision des plexus nerveux péri-artériels. Presse Med.

[B19] Baron R, Maier C (1996). Reflex sympathetic dystrophy: skin blood flow, sympathetic vasoconstrictor reflexes and pain before and after surgical sympathectomy. Pain.

[B20] Kurvers HA, Jacobs MJ, Beuk RJ, Wildenberg FA van den, Kitslaar PJ, Slaaf DW, Reneman RS (1994). Reflex sympathetic dystrophy: result of autonomic denervation?. Clin Sci (Lond).

[B21] Wasner G, Schattschneider J, Heckmann K, Maier C, Baron R (2001). Vascular abnormalities in reflex sympathetic dystrophy (CRPS I): mechanisms and diagnostic value. Brain.

[B22] Bini G, Hagbarth KE, Hynninen P, Wallin BG (1980). Thermoregulatory and rhythm-generating mechanisms governing the sudomotor and vasoconstrictor outflow in human cutaneous nerves. The Journal of physiology.

[B23] Niehof SP, Huygen FJ, Weerd RW van der, Westra M, Zijlstra FJ (2006). Thermography imaging during static and controlled thermoregulation in complex regional pain syndrome type 1: diagnostic value and involvement of the central sympathetic system. Biomed Eng Online.

[B24] Schurmann M, Zaspel J, Lohr P, Wizgall I, Tutic M, Manthey N, Steinborn M, Gradl G (2007). Imaging in early posttraumatic complex regional pain syndrome: a comparison of diagnostic methods. Clin J Pain.

[B25] Wasner G, Heckmann K, Maier C, Baron R (1999). Vascular abnormalities in acute reflex sympathetic dystrophy (CRPS I): complete inhibition of sympathetic nerve activity with recovery. Arch Neurol.

[B26] Kurvers HA, Jacobs MJ, Beuk RJ, Wildenberg FA Van den, Kitslaar PJ, Slaaf DW, Reneman RS (1995). Reflex sympathetic dystrophy: evolution of microcirculatory disturbances in time. Pain.

[B27] Ubbink DT, Jacobs MJ, Slaaf DW, Tangelder GJ, Reneman RS (1992). Microvascular reactivity differences between the two legs of patients with unilateral lower limb ischaemia. European journal of vascular surgery.

[B28] Wasner G, Drummond PD, Birklein F, Baron R, Harden R, Baron R, Jänig (2001). The role of the sympathetic nervous system in autonomic disturbances and 'sympathetically maintained pain' before and after surgical sympathectomy. Progress in Pain Research and Management.

[B29] Drummond PD (2001). Mechanism of complex regional pain syndrome: no longer excessive sympathetic outflow?. Lancet.

[B30] Wasner G, Schattschneider J, Binder A, Baron R (2003). Complex regional pain syndrome - diagnostic, mechanisms, CNS involvement and therapy. Spinal Cord.

[B31] Arnold JM, Teasell RW, MacLeod AP, Brown JE, Carruthers SG (1993). Increased venous alpha-adrenoceptor responsiveness in patients with reflex sympathetic dystrophy. Annals of internal medicine.

[B32] Toda K, Muneshige H, Asou T, Harada T, Okazaki M, Tachiki N, Horie N, Cheng W, Nakamura S (2006). Basal blood flow in complex regional pain syndrome does not necessarily indicate vasoconstrictor nerve activity. Clin J Pain.

[B33] Birklein F, Weber M, Ernst M, Riedl B, Neundorfer B, Handwerker HO (2000). Experimental tissue acidosis leads to increased pain in complex regional pain syndrome (CRPS). Pain.

[B34] Koban M, Leis S, Schultze-Mosgau S, Birklein F (2003). Tissue hypoxia in complex regional pain syndrome. Pain.

[B35] Oyen WJ, Arntz IE, Claessens RM, Meer JW Van der, Corstens FH, Goris RJ (1993). Reflex sympathetic dystrophy of the hand: an excessive inflammatory response?. Pain.

[B36] Vartiainen NV, Kirveskari E, Forss N (2008). Central processing of tactile and nociceptive stimuli in complex regional pain syndrome. Clin Neurophysiol.

[B37] Maihofner C, Handwerker HO, Neundorfer B, Birklein F (2004). Cortical reorganization during recovery from complex regional pain syndrome. Neurology.

[B38] Moseley GL (2004). Graded motor imagery is effective for long-standing complex regional pain syndrome: a randomised controlled trial. Pain.

[B39] Moseley GL, Brhyn L, Ilowiecki M, Solstad K, Hodges PW (2003). The threat of predictable and unpredictable pain. Aust J Physiother.

[B40] Huge V, Lauchart M, Forderreuther S, Kaufhold W, Valet M, Azad SC, Beyer A, Magerl W (2008). Interaction of hyperalgesia and sensory loss in complex regional pain syndrome type I (CRPS I). PLoS ONE.

[B41] Janig W, Baron R (2002). Complex regional pain syndrome is a disease of the central nervous system. Clin Auton Res.

[B42] Endemann DH, Schiffrin EL (2004). Endothelial dysfunction. J Am Soc Nephrol.

[B43] Alonso D, Radomski MW (2003). Nitric oxide, platelet function, myocardial infarction and reperfusion therapies. Heart Fail Rev.

[B44] Alonso D, Radomski MW (2003). The nitric oxide-endothelin-1 connection. Heart Fail Rev.

[B45] Kinlay S, Selwyn AP, Delagrange D, Creager MA, Libby P, Ganz P (1996). Biological mechanisms for the clinical success of lipid-lowering in coronary artery disease and the use of surrogate end-points. Current opinion in lipidology.

[B46] Moncada S, Higgs EA, Vane JR (1977). Human arterial and venous tissues generate prostacyclin (prostaglandin x), a potent inhibitor of platelet aggregation. Lancet.

[B47] De Caterina R, Massaro M, Libby P, De Caterina R, Libby P (2007). Endothelial functions and dysfunctions. Endothelial dysfunctions and vascular disease.

[B48] Yanagisawa M, Kurihara H, Kimura S, Tomobe Y, Kobayashi M, Mitsui Y, Yazaki Y, Goto K, Masaki T (1988). A novel potent vasoconstrictor peptide produced by vascular endothelial cells. Nature.

[B49] Verhaar MC, Strachan FE, Newby DE, Cruden NL, Koomans HA, Rabelink TJ, Webb DJ (1998). Endothelin-A receptor antagonist-mediated vasodilatation is attenuated by inhibition of nitric oxide synthesis and by endothelin-B receptor blockade. Circulation.

[B50] Miyauchi T, Masaki T (1999). Pathophysiology of endothelin in the cardiovascular system. Annual review of physiology.

[B51] Kinlay S, Ganz P, De Caterina R, Libby P (2007). Endothelial vasodilatory dysfunction. Endothelial dysfunctions and vascular disease.

[B52] Cardillo C, Kilcoyne CM, Cannon RO, Panza JA (2000). Interactions between nitric oxide and endothelin in the regulation of vascular tone of human resistance vessels in vivo. Hypertension.

[B53] Panza JA, Quyyumi AA, Brush JE, Epstein SE (1990). Abnormal endothelium-dependent vascular relaxation in patients with essential hypertension. The New England journal of medicine.

[B54] Kinlay S, Libby P, Ganz P (2001). Endothelial function and coronary artery disease. Current opinion in lipidology.

[B55] Carr AC, Zhu BZ, Frei B (2000). Potential antiatherogenic mechanisms of ascorbate (vitamin C) and alpha-tocopherol (vitamin E). Circ Res.

[B56] Joannides R, Haefeli WE, Linder L, Richard V, Bakkali EH, Thuillez C, Luscher TF (1995). Nitric oxide is responsible for flow-dependent dilatation of human peripheral conduit arteries in vivo. Circulation.

[B57] Kooijman M, Thijssen DH, de Groot PC, Bleeker MW, van Kuppevelt HJ, Green DJ, Rongen GA, Smits P, Hopman MT (2008). Flow-mediated dilatation in the superficial femoral artery is nitric oxide mediated in humans. The Journal of physiology.

[B58] Perticone F, Ceravolo R, Pujia A, Ventura G, Iacopino S, Scozzafava A, Ferraro A, Chello M, Mastroroberto P, Verdecchia P (2001). Prognostic significance of endothelial dysfunction in hypertensive patients. Circulation.

[B59] Furchgott RF, Zawadzki JV (1980). The obligatory role of endothelial cells in the relaxation of arterial smooth muscle by acetylcholine. Nature.

[B60] Ludmer PL, Selwyn AP, Shook TL, Wayne RR, Mudge GH, Alexander RW, Ganz P (1986). Paradoxical vasoconstriction induced by acetylcholine in atherosclerotic coronary arteries. The New England journal of medicine.

[B61] Celermajer DS, Sorensen KE, Gooch VM, Spiegelhalter DJ, Miller OI, Sullivan ID, Lloyd JK, Deanfield JE (1992). Non-invasive detection of endothelial dysfunction in children and adults at risk of atherosclerosis. Lancet.

[B62] Kuvin JT, Patel AR, Sliney KA, Pandian NG, Sheffy J, Schnall RP, Karas RH, Udelson JE (2003). Assessment of peripheral vascular endothelial function with finger arterial pulse wave amplitude. American heart journal.

[B63] Ganz P, Vita JA (2003). Testing endothelial vasomotor function: nitric oxide, a multipotent molecule. Circulation.

[B64] Duman I, Sanal HT, Dincer K, Kalyon TA (2008). Assessment of endothelial function in complex regional pain syndrome type I. Rheumatology international.

[B65] Park JB, Charbonneau F, Schiffrin EL (2001). Correlation of endothelial function in large and small arteries in human essential hypertension. Journal of hypertension.

[B66] Gorodkin R, Moore T, Herrick A (2004). Assessment of endothelial function in complex regional pain syndrome type I using iontophoresis and laser Doppler imaging. Rheumatology (Oxford, England).

[B67] Schattschneider J, Hartung K, Stengel M, Ludwig J, Binder A, Wasner G, Baron R (2006). Endothelial dysfunction in cold type complex regional pain syndrome. Neurology.

[B68] Dayan L, Salman S, Norman D, Vatine JJ, Calif E, Jacob G (2008). Exaggerated vasoconstriction in complex regional pain syndrome-1 is associated with impaired resistance artery endothelial function and local vascular reflexes. J Rheumatol.

[B69] Mogilevsky M, Janig W, Baron R, Harden RN (2007). Complex regional pain syndrome-a multifaceted disorder requiring multidimensional care: case study. J Pain.

[B70] Woolf CJ (2004). Pain: moving from symptom control toward mechanism-specific pharmacologic management. Annals of internal medicine.

[B71] Janig W, Baron R (2006). Is CRPS I a neuropathic pain syndrome?. Pain.

[B72] Gibbs GF, Drummond PD, Finch PM, Phillips JK (2008). Unravelling the Pathophysiology of Complex Regional Pain Syndrome: Focus on Sympathetically Maintained Pain. Clin Exp Pharmacol Physiol.

[B73] Xanthos DN, Coderre TJ (2008). Sympathetic Vasoconstrictor Antagonism and Vasodilatation Relieve Mechanical Allodynia in Rats With Chronic Postischemia Pain. J Pain.

[B74] Price DD, Long S, Huitt C (1992). Sensory testing of pathophysiological mechanisms of pain in patients with reflex sympathetic dystrophy. Pain.

[B75] Ali Z, Raja SN, Wesselmann U, Fuchs PN, Meyer RA, Campbell JN (2000). Intradermal injection of norepinephrine evokes pain in patients with sympathetically maintained pain. Pain.

[B76] Torebjork E, Wahren L, Wallin G, Hallin R, Koltzenburg M (1995). Noradrenaline-evoked pain in neuralgia. Pain.

[B77] Baron R, Schattschneider J, Binder A, Siebrecht D, Wasner G (2002). Relation between sympathetic vasoconstrictor activity and pain and hyperalgesia in complex regional pain syndromes: a case-control study. Lancet.

[B78] Drummond PD, Finch PM, Skipworth S, Blockey P (2001). Pain increases during sympathetic arousal in patients with complex regional pain syndrome. Neurology.

[B79] Kaplan R, Claudio M, Kepes E, Gu XF (1996). Intravenous guanethidine in patients with reflex sympathetic dystrophy. Acta Anaesthesiol Scand.

[B80] Drummond PD (2004). Involvement of the sympathetic nervous system in complex regional pain syndrome. The international journal of lower extremity wounds.

[B81] Drummond PD, Finch PM (2004). Persistence of pain induced by startle and forehead cooling after sympathetic blockade in patients with complex regional pain syndrome. J Neurol Neurosurg Psychiatry.

[B82] Cepeda MS, Carr DB, Lau J (2005). Local anaesthetic sympathic blockade for complex regional pain syndrome. Cochrane Database ofSystematic Reviews.

[B83] Sudeck P (1900). Über die acute entzündliche Knochenatrophie. Archiv für klinische Chirurgie.

[B84] Sudeck P (1901). Über die akute (reflektorische) Knochenatrophie nach Entzündungen und Verletzungen an den Extremitäten und ihre klinischen Erscheinungen. Fortschritte auf dem Gebiete der Röntgenstrahlen.

[B85] Kozin F, Genant HK, Bekerman C, McCarty DJ (1976). The reflex sympathetic dystrophy syndrome. II. Roentgenographic and scintigraphic evidence of bilaterality and of periarticular accentuation. The American journal of medicine.

[B86] Birklein F, Schmelz M, Schifter S, Weber M (2001). The important role of neuropeptides in complex regional pain syndrome. Neurology.

[B87] Beek WJ van de, Remarque EJ, Westendorp RG, van Hilten JJ (2001). Innate cytokine profile in patients with complex regional pain syndrome is normal. Pain.

[B88] Wesseldijk F, Huygen FJ, Heijmans-Antonissen C, Niehof SP, Zijlstra FJ (2007). Tumor necrosis factor-alpha and interleukin-6 are not correlated with the characteristics of Complex Regional Pain Syndrome type 1 in 66 patients. Eur J Pain.

[B89] Groeneweg JG, Huygen FJ, Heijmans-Antonissen C, Niehof S, Zijlstra FJ (2006). Increased endothelin-1 and diminished nitric oxide levels in blister fluids of patients with intermediate cold type complex regional pain syndrome type 1. BMC musculoskeletal disorders.

[B90] Munnikes RJ, Muis C, Boersma M, Heijmans-Antonissen C, Zijlstra FJ, Huygen FJ (2005). Intermediate stage complex regional pain syndrome type 1 is unrelated to proinflammatory cytokines. Mediators Inflamm.

[B91] Wesseldijk F, Huygen FJ, Heijmans-Antonissen C, Niehof SP, Zijlstra FJ (2008). Six years follow-up of the levels of TNF-alpha and IL-6 in patients with complex regional pain syndrome type 1. Mediators Inflamm.

[B92] Blaisdell FW (2002). The pathophysiology of skeletal muscle ischemia and the reperfusion syndrome: a review. Cardiovascular surgery (London, England).

[B93] Coderre TJ, Xanthos DN, Francis L, Bennett GJ (2004). Chronic post-ischemia pain (CPIP): a novel animal model of complex regional pain syndrome-type I (CRPS-I; reflex sympathetic dystrophy) produced by prolonged hindpaw ischemia and reperfusion in the rat. Pain.

[B94] Millecamps M, Coderre TJ (2008). Rats with chronic post-ischemia pain exhibit an analgesic sensitivity profile similar to human patients with complex regional pain syndrome - type I. European journal of pharmacology.

[B95] Laferriere A, Millecamps M, Xanthos DN, Xiao WH, Siau C, de Mos M, Sachot C, Ragavendran JV, Huygen FJ, Bennett GJ (2008). Cutaneous tactile allodynia associated with microvascular dysfunction in muscle. Mol Pain.

[B96] Xanthos DN, Bennett GJ, Coderre TJ (2008). Norepinephrine-induced nociception and vasoconstrictor hypersensitivity in rats with chronic post-ischemia pain. Pain.

[B97] Zollinger PE, Tuinebreijer WE, Breederveld RS, Kreis RW (2007). Can vitamin C prevent complex regional pain syndrome in patients with wrist fractures? A randomized, controlled, multicenter dose-response study. J Bone Joint Surg Am.

[B98] Zollinger PE, Tuinebreijer WE, Kreis RW, Breederveld RS (1999). Effect of vitamin C on frequency of reflex sympathetic dystrophy in wrist fractures: a randomised trial. Lancet.

[B99] Goris RJ (1998). Reflex sympathetic dystrophy: model of a severe regional inflammatory response syndrome. World J Surg.

[B100] Goris RJ, Dongen LM, Winters HA (1987). Are toxic oxygen radicals involved in the pathogenesis of reflex sympathetic dystrophy?. Free Radic Res Commun.

[B101] Zuurmond WW, Langendijk PN, Bezemer PD, Brink HE, de Lange JJ, van loenen AC (1996). Treatment of acute reflex sympathetic dystrophy with DMSO 50% in a fatty cream. Acta Anaesthesiol Scand.

[B102] Perez RS, Pragt E, Geurts J, Zuurmond WW, Patijn J, van Kleef M (2008). Treatment of Patients With Complex Regional Pain Syndrome Type I With Mannitol: A Prospective, Randomized, Placebo-Controlled, Double-Blinded Study. J Pain.

[B103] Perez RS, Zuurmond WW, Bezemer PD, Kuik DJ, van Loenen AC, de Lange JJ, Zuidhof AJ (2003). The treatment of complex regional pain syndrome type I with free radical scavengers: a randomized controlled study. Pain.

[B104] Schattschneider J, Binder A, Siebrecht D, Wasner G, Baron R (2006). Complex regional pain syndromes: the influence of cutaneous and deep somatic sympathetic innervation on pain. Clin J Pain.

[B105] Geertzen JH, Dijkstra PU, Groothoff JW, ten Duis HJ, Eisma WH (1998). Reflex sympathetic dystrophy of the upper extremity--a 5.5-year follow-up. Part II. Social life events, general health and changes in occupation. Acta Orthop Scand Suppl.

[B106] Geertzen JH, Dijkstra PU, van Sonderen EL, Groothoff JW, ten Duis HJ, Eisma WH (1998). Relationship between impairments, disability and handicap in reflex sympathetic dystrophy patients: a long-term follow-up study. Clin Rehabil.

[B107] Galer BS, Jensen M (1999). Neglect-like symptoms in complex regional pain syndrome: results of a self-administered survey. J Pain Symptom Manage.

[B108] McCabe CS, Shenker N, Lewis J, Blake DR (2005). Impaired self-perception of the hand in complex regional pain syndrome (CRPS) [S. Forderreuther, U. Sailer, A. Straube, Pain 2004; 110:756-761]. Pain.

[B109] Forderreuther S, Sailer U, Straube A (2004). Impaired self-perception of the hand in complex regional pain syndrome (CRPS). Pain.

[B110] Lewis JS, Kersten P, McCabe CS, McPherson KM, Blake DR (2007). Body perception disturbance: a contribution to pain in complex regional pain syndrome (CRPS). Pain.

[B111] Ushida T, Willis WD (2001). Changes in dorsal horn neuronal responses in an experimental wrist contracture model. J Orthop Sci.

[B112] Butler SH, Norman R, Harden RBaWJ (2001). Disuse and CRPS. Complex Regional Pain Syndrome, Progress in Pain Research and Management.

[B113] Guo TZ, Offley SC, Boyd EA, Jacobs CR, Kingery WS (2004). Substance P signaling contributes to the vascular and nociceptive abnormalities observed in a tibial fracture rat model of complex regional pain syndrome type I. Pain.

[B114] Tahmoush AJ, Schwartzman RJ, Hopp JL, Grothusen JR (2000). Quantitative sensory studies in complex regional pain syndrome type 1/RSD. Clin J Pain.

[B115] Nelson DV, Turk DC, Gatchel R (2002). Treating patients with complex regional pain syndrome. Psychological approaches to pain management A practitioner's handbook.

[B116] de Jong JR, Vlaeyen JW, Onghena P, Cuypers C, den Hollander M, Ruijgrok J (2005). Reduction of pain-related fear in complex regional pain syndrome type I: the application of graded exposure in vivo. Pain.

[B117] Rommel O, Willweber-Strumpf A, Wagner P, Surall D, Malin JP, Zenz M (2005). [Psychological abnormalities in patients with complex regional pain syndrome (CRPS)] Psychische Veranderungen bei Patienten mit komplexem regionalem Schmerzsyndrom (CRPS). Schmerz.

[B118] Laan L van der, van Spaendonck K, Horstink MW, Goris RJ (1999). The Symptom Checklist-90 Revised questionnaire: no psychological profiles in complex regional pain syndrome-dystonia. J Pain Symptom Manage.

[B119] de Mos M, Huygen FJ, Dieleman JP, Koopman JS, Stricker BH, Sturkenboom MC (2008). Medical history and the onset of complex regional pain syndrome (CRPS). Pain.

[B120] Krzyzanowska K, Mittermayer F, Krugluger W, Schnack C, Hofer M, Wolzt M, Schernthaner G (2006). Asymmetric dimethylarginine is associated with macrovascular disease and total homocysteine in patients with type 2 diabetes. Atherosclerosis.

[B121] Yuen KC, Baker NR, Rayman G (2002). Treatment of chronic painful diabetic neuropathy with isosorbide dinitrate spray: a double-blind placebo-controlled cross-over study. Diabetes Care.

[B122] Ezri T, Susmallian S (2003). Topical nifedipine vs. topical glyceryl trinitrate for treatment of chronic anal fissure. Dis Colon Rectum.

[B123] Zuberi BF, Rajput MR, Abro H, Shaikh SA (2000). A randomized trial of glyceryl trinitrate ointment and nitroglycerin patch in healing of anal fissures. International journal of colorectal disease.

[B124] Paoloni JA, Appleyard RC, Nelson J, Murrell GA (2003). Topical nitric oxide application in the treatment of chronic extensor tendinosis at the elbow: a randomized, double-blinded, placebo-controlled clinical trial. Am J Sports Med.

[B125] Badala H, Toledo S (2002). Tratamiento de la Distrofia Simpatico Refleja (DSR). Con nitroglicerina transdermica. Archivos de Medicina Interna Volumen.

[B126] Groeneweg G, Niehof S, Wesseldijk F, Huygen FJ, Zijlstra FJ (2008). Vasodilative effect of isosorbide dinitrate ointment in complex regional pain syndrome type 1. Clin J Pain.

[B127] Groeneweg JG, Huygen FJ, Niehof S, Wesseldijk F, Bussmann JB, Schasfoort FC, Stronks DL, Zijlstra FJ (2009). No Recovery of Cold Complex Regional Pain Syndrome after Transdermal Isosorbide Dinitrate. A Small Controlled Trial. Journal of Pain and Symptom Management.

[B128] Lue TF (2000). Erectile dysfunction. The New England journal of medicine.

[B129] Groeneweg G, Huygen FJ, Niehof SP, Wesseldijk F, Bussmann JB, Schasfoort FC, Stronks DL, Zijlstra FJ (2008). Effect of tadalafil on blood flow, pain, and function in chronic cold Complex Regional Pain Syndrome: a randomized controlled trial. BMC musculoskeletal disorders.

[B130] Niehof SP, Huygen FJ, Stronks DL, Klein J, Zijlstra FJ (2007). Reliability of observer assessment of thermographic images in complex regional pain syndrome type 1. Acta orthopaedica Belgica.

[B131] Niehof SP, Beerthuizen A, Huygen FJ, Zijlstra FJ (2008). Using skin surface temperature to differentiate between complex regional pain syndrome type 1 patients after a fracture and control patients with various complaints after a fracture. Anesth Analg.

[B132] Krumova EK, Frettloh J, Klauenberg S, Richter H, Wasner G, Maier C (2008). Long-term skin temperature measurements - a practical diagnostic tool in complex regional pain syndrome. Pain.

[B133] Kurvers HA, Jacobs MJ, Beuk RJ, Wildenberg FA van den, Kitslaar PJ, Slaaf DW, Reneman RS (1996). The spinal component to skin blood flow abnormalities in reflex sympathetic dystrophy. Arch Neurol.

[B134] Prough DS, McLeskey CH, Poehling GG, Koman LA, Weeks DB, Whitworth T, Semble EL (1985). Efficacy of oral nifedipine in the treatment of reflex sympathetic dystrophy. Anesthesiology.

[B135] Muizelaar JP, Kleyer M, Hertogs IA, DeLange DC (1997). Complex regional pain syndrome (reflex sympathetic dystrophy and causalgia): management with the calcium channel blocker nifedipine and/or the alpha-sympathetic blocker phenoxybenzamine in 59 patients. Clin Neurol Neurosurg.

[B136] Ellrodt AG, Singh BN (1983). Clinical applications of slow channel blocking compounds. Pharmacol Ther.

[B137] Noll G, Luscher TF (1998). Comparative pharmacological properties among calcium channel blockers: T-channel versus L-channel blockade. Cardiology.

[B138] Pache M, Schwarz HA, Kaiser HJ, Wuest P, Kloti M, Dubler B, Flammer J (2002). Elevated plasma endothelin-1 levels and vascular dysregulation in patients with rheumatoid arthritis. Med Sci Monit.

[B139] El Melegy NT, Ali ME, Awad EM (2005). Plasma levels of endothelin-1, angiotensin II, nitric oxide and prostaglandin E in the venous and cavernosal blood of patients with erectile dysfunction. BJU Int.

[B140] Menger MD, Pelikan S, Steiner D, Messmer K (1992). Microvascular ischemia-reperfusion injury in striated muscle: significance of "reflow paradox". Am J Physiol.

[B141] Bej MD, Schwartzman RJ (1991). Abnormalities of cutaneous blood flow regulation in patients with reflex sympathetic dystrophy as measured by laser Doppler fluxmetry. Arch Neurol.

[B142] Schurmann M, Gradl G, Furst H (1996). A standardized bedside test for assessment of peripheral sympathetic nervous function using laser Doppler flowmetry. Microvascular research.

[B143] Schurmann M, Gradl G, Andress HJ, Furst H, Schildberg FW (1999). Assessment of peripheral sympathetic nervous function for diagnosing early post-traumatic complex regional pain syndrome type I. Pain.

[B144] Singh B, Moodley J, Shaik AS, Robbs JV (2003). Sympathectomy for complex regional pain syndrome. J Vasc Surg.

[B145] Gradl G, Beyer A, Azad S, Schurmann M (2005). [Evaluation of sympathicolysis after continuous brachial plexus analgesia using laser Doppler flowmetry in patients suffering from CRPS I]. Anasthesiol Intensivmed Notfallmed Schmerzther.

[B146] Drummond PD (2004). Involvement of the Sympathetic Nervous System in Complex Regional Pain Syndrome. Lower Extremity Wounds.

[B147] Cooke ED, Harris J, Fleming CE, Steinberg MD, Foster JM (1995). Correlation of pain with temperature and blood-flow changes in the lower limb following chemical lumbar sympathectomy in reflex sympathetic dystrophy. A case report. Int Angiol.

[B148] Bolel K, Hizmetli S, Akyuz A (2006). Sympathetic skin responses in reflex sympathetic dystrophy. Rheumatology international.

